# Team ball sport experience minimizes sex difference in visual attention

**DOI:** 10.3389/fpsyg.2022.987672

**Published:** 2022-10-13

**Authors:** Peng Jin, Zhigang Ge, Tieming Fan

**Affiliations:** ^1^Department of Physical Education, Southeast University, Nanjing, China; ^2^Nanjing No. 9 Middle School, Nanjing, China

**Keywords:** visual attention, sex differences, team ball sport, multiple-object tracking, basketball player, college students

## Abstract

Simultaneous tracking of the position of the ball and player locations and activities places high demands on visual attention in team ball sport athletes. Owing to their extensive sports training, these athletes may be expected to exhibit visual attention skills superior to non-athletes; however, the results of studies examining this are inconsistent. Thus, the first aim of this study was to assess the impact of participating in a team ball sport on visual attention. There is limited empirical evidence indicating a sex difference in visual attention, and few studies have reported on visual attention in male and female athletes. Thus, the second aim of this study was to determine whether team ball sport experience affected any sex differences in visual attention. In total, 44 highly skilled basketball players—22 men (mean age: 21.86 ± 2.15 years) with a mean (SD) of 8.46 (2.92) years training experience and 22 women (mean age: 21.32 ± 1.58 years) with a mean (SD) of 8.22 (2.44) years of training experience—and 44 non-athletic undergraduate college students—22 men (mean age: 21.62 ± 1.88 years) and 22 women (mean age: 21.55 ± 1.72 years)—were recruited and completed this study. Visual attention was measured by using the multiple object tracking (MOT) task. Skilled basketball players showed superior tracking accuracy to non-athletic college students on the MOT task. A significant sex difference was found only among the non-athletic college students, with better tracking accuracy for men than for women on the MOT task. By contrast, no significant sex difference was observed among the skilled basketball players for tracking accuracy on the MOT task. These findings indicated that team ball sport training may enhance visual attention as assessed by tracking accuracy. Given that the male and female basketball players in this study had similar training experience and game performance demands, long-term team ball sport experience appeared to minimize the sex difference in visual attention found among non-athletic students.

## Introduction

Visual attention plays a crucial role in every task involving perceiving and acting, especially participating in sports. Visual attention underlies perceptual skills in sports (Ward and Williams, 2003), it may affect the performance of players and thus the match outcome. In sports situations, athletes must rapidly and accurately extract visual information to make a correct decision (Walsh, 2014). This ability is particularly critical in team ball sports, in which athletes are required to track the position of the ball while simultaneously monitoring the activities and locations of all the athletes on the field (Howard et al., 2018); a task that is likely extremely demanding for visual attention as well as one that requires the flexibility of the motor system (Howard et al., 2018).

A number of studies have examined the relationship between team ball sport participation and visual attention. Many studies have demonstrated that compared with non-athletes, athletes participating in team ball sports exhibit superior visual attention (Cer Ea Tti et al., 2009; Heloisa et al., 2013; Notarnicola et al., 2014). It is widely accepted that an enriched environment leads to plasticity changes in the brain (May, 2011). Research also suggests that sports training may have positive transfer effects on relevant cognitive functioning (Heppe et al., 2016), particularly for visual attention (Zhang et al., 2021). Hence, it is reasonable to conclude that athletes will exhibit superior visual attention performance owing to their extensive sports training (Alves et al., 2013). However, not all the empirical evidence is consistent with this conclusion; some studies have found no statistical difference between athletes and non-athletes. Memmert et al. (2009) reported that experts in team sports did not perform better in visual attention tasks. They showed that there were no individual differences in the performance of a visual attention task between basketball players and non-athlete college students. A potential reason for these discrepant findings may be in the procedure of the paradigm itself (Meyerhoff et al., 2017), group differences (i.e., expertise effects) were modulated by attentional load, which “expert advantage” cannot be displayed under low task difficulty (Qiu et al., 2018; Jin et al., 2020). Therefore, the main goal of the present study was to assess the differences in visual attention between athletes and non-athletes to investigate whether sports activities influence visual attention.

A common finding in the general population is that males perform better than females on tasks measuring visuospatial skills owing to sociocultural and biological factors (Collins and Kimura, 1997; Ryan et al., 2004; Notarnicola et al., 2014). However, Merritt et al. (2007) showed that there were no significant differences between females and males in visual selective attention. There is limited empirical evidence indicating sex differences in visual attention, and few studies in the sports literature have reported on visual attention in male and female athletes, highlighting the need for more research in this area. One study indicated that male dynamic sports athletes were better than female dynamic sports athletes at orienting attention in a covert orienting task (Lum et al., 2002), but the results of another study indicated a lack of sex differences in the performance of a visual-spatial task among volleyball players and among tennis players (Notarnicola et al., 2014). Sex appears to be a crucial component influencing cognitive function, and it interacts with the experience of sport activities. Therefore, although evidence is accumulating regarding sex differences in visual attention among athletes and non-athletes, this remains an area requiring further investigation.

An important study demonstrated that sport experience reduce sex effects in visual attention (Alves et al., 2013), which showed that female and male volleyball athletes exhibited similar selective visual attention in the Flanker task, and female volleyball athletes were just as good as male volleyball athletes in visual attention when they perform a Change Detection task. However, non-athletes adult control group have shown that men consistently perform better than women on measures of the two visual attention tasks. Similarly, the results of another study that examined gender differences in a visual spatial attention task suggested that Male volleyball player were no better than female volleyball player and indicated that better performance in males than females in a visual spatial attention task among non-sport activity group (Notarnicola et al., 2014). It is important to determine whether sex differences may be minimized or even eliminated, it can give us clues to be used in teaching and training, such as the female player can perform the same male tactical schemes. If a sex difference in visual attention can be minimized by appropriate and timely training, female athletes may be considered on par with male athletes with regard to this aspect of the sport. Research assessing the influence of sports activities on sex differences in visual attention is at an early stage and currently lacks compelling evidence. On this ground, we aim to examine weather sport experience affected sex differences on visual attention.

One well-established paradigm used to investigate visual attention is multiple object tracking (MOT), which was originated by Pylyshyn and Storm (1988). In a classic MOT task, participants visually track the positions of multiple independent target items moving among identical distractors (Terry and Trick, 2021). The MOT task has proven to be one of the most useful and popular tools in the study of visual attention, which has been employed to assess aspects of sustained, distributed, and selective attention skills (Koldewyn et al., 2013; Meyerhoff et al., 2017; Meyerhoff and Papenmeier, 2020). This study investigated players from team-ball (i.e., basketball players) sports because visual attention plays a key role in these types of games. Players need to simultaneously pay attention not only to the spatial position of the ball and to the court but also to the movement and position of teammates and opponents (Zariæ et al., 2018). Therefore, it is reasonable to suggest that professional players of team ball sports may exhibit superior MOT performances owing to their extensive sports training (Alves et al., 2013). However, results from another study indicated that players of team ball sports with more than 10 years of training did not show better tracking performance compared to non-athletes in the MOT task (Memmert et al., 2009). Thus, the ability to estimate a player’s capability to perform in measures that are related to team success would prove beneficial for cognitive training, player recruitment and needs analysis (Mangine et al., 2014). In addition, sex differences in the MOT task have not been explored systematically in the sports literature, and little is known about whether sports activities may eliminate sex differences in the MOT task.

Therefore, the present study had two objectives. The first aim was to assess the impact of participating in a team ball sport on the development of visual attention. Derived from this aim, the second but main goal of this study was to investigate whether team ball sport experience eliminated sex differences in visual attention. Our hypotheses were twofold. First, we hypothesized that athletes involved in team ball sports would exhibit tracking performance superior to that of non-athletes in the MOT task. Second, we hypothesized that sex differences for tracking performance in the MOT task would be observed only among non-athletes, and that team ball sport activities would minimize these sex differences in athletes.

## Materials and methods

### Participants

Calculation of the required sample size was performed using G*Power 3.1.9. With an effect size of 0.35, a desired power of 0.90, and an alpha level of 0.05, we determined that the sample size would need to be 22 per group. Thus, a total of 44 basketball players were recruited from six China University Basketball Association (CUBA) teams. The players comprised 22 men (age: 21.86 ± 2.15 years) with 8.46 (SD = 2.92) years of training experience, and 12.70 (SD = 2.36) training hours per week as well as 22 women (age: 21.32 ± 1.58 years), with 8.22 (SD = 2.44) years of training experience and 12.62 (SD = 2.15) training hours per week. All basketball players were first- and second-level national athletes. The non-athlete group—composed of 22 men (age: 21.62 ± 1.88 years) and 22 women (age: 21.55 ± 1.72 years)—were undergraduate students who had never participated in basketball training or any other team ball sports. All participants reported normal or corrected-to-normal levels of visual function and were right-handed. The participants were asked to avoid staying up late (to remain awake past one’s usual or required bedtime) at night to ensure good sleep quality. All recruited basketball players and college students completed the study. The study protocol was approved by the ethics committee of Shanghai University of Sport (No. 2015003SUS). Informed consent (regarding the purpose of the study, task description, and the responsibilities, obligations, and rights of the participants) was obtained in writing prior to the test session. Each participant who completed the research was paid $10 in return for their time. None of the participants had solved a MOT test before or had been trained in MOT.

### Task stimuli, apparatus, and procedure

**FIGURE 1:**
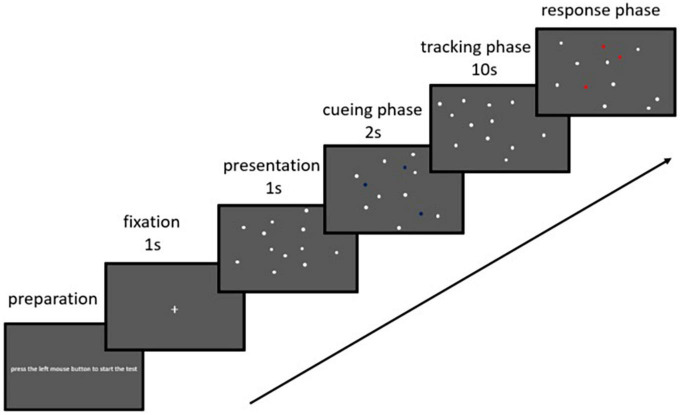
A schematic diagram of the visual stimuli in one trial of the multiple object tracking task. Twelve filled white circles are presented. In each trial, three of the circles turn blue and flicker for 2 s before turning back to white. The blue flickering circles indicate the targets for that trial. All 12 circles then move 10°/s for a 10-s tracking period. When the circles stop moving, the participants use a mouse to click over their choices for the target circles.

The experiment was conducted using a Dell G5 15 laptop computer with MATLAB R2016a and Psychtoolbox 3 software. Visual stimuli were presented on a 15.6-inch (39.62 cm) monitor with a 1,920 × 1,080 resolution and a 60-Hz refresh rate. The participants were seated at a distance of 55 cm from the screen and tested individually in a quiet room and with low lighting. At the beginning of the task, the preparation screen was presented, which said “press the left mouse button to start the test” (see Figure 1). A white fixation cross (+) was presented for 1,000 ms at the center of a gray background (visual field, 37.98° × 21.0°) at the beginning of each trail, followed by 12 white filled circles (0.65° diameter) for 1,000 ms. In each trail, three filled circles were highlighted blue and flickered three times for 2 s to mark them as targets for the trial. After that, the target circles returned to white so that no cue remained to discriminate them from the distractors. Next, all 12 filled circles moved in random directions at a constant speed of 10°/s, with the movement of each circle being affected only by collisions. After 10 s, the circles were frozen in place. The participants were instructed to point out the targets by pressing a mouse button. Based on previous research (Memmert et al., 2009), we did not require the participants to respond quickly to ensure tracking accuracy during the experiment. The response also triggered the start of the next trial.

The experimental procedure consisted of 40 trials divided into two blocks, with a rest period of 5 min between the blocks. The task took approximately 15 min to complete. Participants received five practice trials to ensure that they were familiar with the task procedure before the formal testing session.

### Statistical analysis

Data were recorded and collected by MATLAB R2016a software. IBM SPSS, version 23.0, software was used to calculate the tracking accuracy and to conduct statistical analyses. A univariate general linear model (UNIANOVA) analysis was carried out, with sex (male, female) and group (basketball player, student) as independent variables, and tracking accuracy on the MOT task as a dependent variable. Further analyses were conducted using the simple effects test for significant interactions. Simple effects were analyzed by adding the following to the software analysis:/EMMEANS = TABLES (sex*group) COMPARE (group) ADJ (LSD),/EMMEANS = TABLES (group*sex) COMPARE (gender) ADJ (LSD). Effect size calculations were performed using Cohen’s d and the classifications as follows: 0.2, 0.5, and 0.8 for small, medium, and large effect sizes. An alpha level of 0.05 was considered statistically significant. We calculated tracking accuracy by determining the number of correctly selected targets across all 40 trails for each participant.

## Results

The UNIANOVA analysis of tracking accuracy data showed a significant main effect of sex, *F*_(1, 84)_ = 18.928, *p* < 0.001, η*_*P*_*^2^ = 0.184, and indicated that males (mean = 0.56, SD = 0.10) had significantly better tracking performance than females (mean = 0.49, SD = 0.13). There was also a significant main effect of group, *F*_(1, 84)_ = 83.494, *p* < 0.001, η*_*P*_*^2^ = 0.498, indicating that basketball players (mean = 0.61, SD = 0.09) had significantly better tracking performance than students (mean = 0.45, SD = 0.09). In addition, a significant interaction was observed between sex and group, *F*_(1, 84)_ = 83.494, *p* < 0.001, η*_*P*_*^2^ = 0.498 (see Table 1).

**TABLE 1:** UNIANOVA test of between-subjects effects.

Source	Type III sum of squares	df	Mean square	*F*	Significance level	Partial eta squared
Corrected model	0.729^a^	3	0.243	37.415	0.000	0.572
Intercept	24.476	1	24.476	3766.385	0.000	0.978
Sex	0.123	1	0.123	18.928	0.000	0.184
Group	0.543	1	0.543	83.494	0.000	0.498
Sex × group	0.064	1	0.064	9.822	0.002	0.105
Error	0.546	84	0.006			
Total	25.751	88				
Corrected total	1.275	87				

Dependent variable = tracking accuracy.

^a^*R*^2^ = 0.572 (adjusted *R*^2^ = 0.557).

Further simple effects analyses showed that for sex, male basketball players (mean = 0.62, SD = 0.09) had significantly better tracking performance than male students (mean = 0.51, SD = 0.08), *p* < 0.01, *d* = 1.29, and that female basketball players (mean = 0.60, SD = 0.09) had significantly better tracking performance than female students (mean = 0.38, SD = 0.05), *p* < 0.01, *d* = 3.02. An analysis by group showed that male students (mean = 0.51, SD = 0.08) had significantly better tracking performance than female students (mean = 0.38, SD = 0.05), *p* < 0.01, *d* = 1.94. However, there was no significant difference between male basketball players (mean = 0.62, SD = 0.09) and female basketball players (mean = 0.60, SD = 0.09) (see Table 2).

**TABLE 2:** Sex and group interactions.

					Mean difference	SE	Significance level^a^	95% CI	
								Lower	Upper
***Sex***	Male	Player	Male	Student	0.103*	0.024	0.000	0.055	0.152
	Female	Player	Female	Student	0.211*	0.024	0.000	0.163	0.259
***Group***	Player	Male	Player	Female	0.021	0.024	0.392	−0.027	0.069
	Student	Male	Student	female	0.129*	0.024	0.000	0.080	0.177

CI, confidence interval.

Dependent variable = tracking accuracy.

*The mean difference is significant at the 0.05 level.

^a^Adjustment for multiple comparisons: LSD (equivalent to no adjustments).

## Discussion

The current study was designed (1) to investigate the differences in visual attention between team ball sport athletes and non-athletic college students and (2) to examine whether team ball sport experience affects sex differences in visual attention. Our results provided evidence to support our hypothesis that basketball athletes would show superior performance to non-athletes for tracking accuracy in a MOT task. Our results also showed a significant sex difference in target tracking accuracy on the MOT task in the non-athlete group. By contrast, no sex difference in target tracking accuracy on the MOT task was detected among the basketball players. Our results suggested that team ball sport experience was associated with the development of visual attention, which minimized the sex difference.

Our results demonstrating that the tracking accuracy of basketball players was markedly better than that of non-athletic college students in the MOT task indicated that the effects of team ball sport experience transferred from a sport-specific task to the MOT task. This finding is consistent with those from other studies that found that basketball players exhibit better visual attention than non-athletic university students (Qiu et al., 2019). In team ball sports, players monitor the positions and movements of the ball but also track the positions of opponents and teammates on the field, which is replicated in the requirements of the MOT paradigm. Other studies also showed that superior tracking performance in the MOT task is significantly related to training experience among team-ball players (Faubert, 2013; Fanghui et al., 2018). Hence, there is evidence to support the notion that team ball sport activities may improve visual attention. However, such results are inconsistent with those of Memmert et al. (2009), who found that team ball sport expertise appeared to be unassociated with superior visual attention in expert handball players compared with non-athletes in a MOT task. These discrepant findings are likely attributable to the task difficulty being too easy to probe visual attention for both basketball players and non-athletic college students in the study by Memmert et al. (2009). That study used seven objects in total, with three circles designated as targets and only four distractors, whereas our study used 12 objects with three targets and nine distractors. Tracking difficulty in the MOT task has been shown to raise as the number of distractors increases (Bettencourt and Somers, 2009; Tombu and Seiffert, 2011; Feria, 2012, 2013). Another plausible explanation for the discrepant results is that causality in the opposite direction if people who are skilled in tracking multiple objects are drawn to team ball sport pursuits; similarly, that people who were good at tracking fast objects continued playing team-ball sports (Howard et al., 2018; Jin et al., 2020). On the whole, our results extended those of previous studies and confirmed our hypothesis that team ball sport training confers enhanced visual attention as assessed by increased tracking accuracy on the MOT task.

We also found a significant sex difference in performance in the MOT task among the non-athletic college students, with men showing higher tracking accuracy than women. Our finding is consistent with a previous study showing an effect of sex in a MOT task, with men showing superior tracking performance (Roudaia and Faubert, 2017). Similar results were also observed in other studies using a visual-spatial task, with men showing performances superior to women (Silverman et al., 2007; Notarnicola et al., 2014). These findings may be attributable to sociocultural factors which men participate more sports or video games than women in current society (Eugenie and Jocelyn, 2017) or to the asymmetry and percentage of the principal cranial tissue volumes between the sexes (Gur and Gur, 2017). Our results supported our hypothesis and provided evidence to suggest that among non-athletes, males have a marked advantage over females in multitarget tracking tasks, highlighting the need to control for sex in studies comparing attentional tracking abilities for general population.

Of primary interest to us were our results showing a sex by group interaction. The college students showed a significant sex difference in tracking accuracy on the MOT task. However, as hypothesized, we found no statistically significant sex difference among the team ball sport athletes. To our knowledge, only a few studies have explored whether engaging in team sports minimizes sex differences in visual attention. One study reported that female volleyball athletes and male volleyball athletes exhibited similar visual selective attention capacity (Alves et al., 2013). Another study drew the same conclusion, that there was no statistically significant difference in the performance of a visual-spatial task between male volleyball athletes and female volleyball athletes (Notarnicola et al., 2014). Given that the demands in performance, intensity of competition, and opportunities for training are now relatively similar across competitive men and women’s basketball, if male and female basketball athletes have similar long-term training, the sex difference in visual-spatial ability may become minimized (Ryan et al., 2004; Notarnicola et al., 2014). It is plausible that changes in hormone levels with such training play a role in cognition function development among women. Sex hormones have a high impact on the performance of attention tasks (Holländer et al., 2005), with women showing a greater benefit from increasing androgen levels than men (Lord and Garrison, 1998). Our finding that a sex difference was detected for tracking accuracy on the MOT task only in the non-athlete group—not in the athlete group—suggests that team ball sport experience may minimize the sex difference in visual attention.

The current study has a few limitations. One major shortcoming was that the absence of examining whether discriminates less-skilled vs. expert athletes. These types of studies will provide a greater understanding of whether visual attention in non-domain specific tasks, such as the MOT task used in this study, results in fine-grained differences between levels of expertise. Furthermore, we didn’t investigate whether superior performance in non-domain specific MOT tasks is associated with superior performance in domain-specific decision-making tasks, such as the recognition paradigms and pattern recall (Gorman et al., 2011, 2013). Further investigation of this type will provide a deeper understanding of the utility of MOT tasks to understand sport expertise. Also, the participants in this study are not representative of all team ball sports. Future studies should consider recruiting participants from a range of team ball sports, including soccer, rugby, and volleyball. Lastly, it is just a quasi-experimental research design, not a Randomized Controlled trial. It is impossible for our paradigm to assign subjects randomly, it is always possible that team-ball players are not better in tracking faster objects, but that people who were good at tracking fast objects continued playing team-ball sport. A longitudinal design is necessary to investigate the change in visual attention skills longitudinally in basketball players over time in the further study. Despite these limitations, the present study is the first, to our knowledge, to show that team ball sport experience can minimize the sex difference in visual attention.

In conclusion, the results of the present research demonstrate that team ball sport players showed higher tracking accuracy than non-athletic college students in a MOT task. In addition, in the non-athlete college group, male students exhibited tracking performance superior to female students; by contrast, there was no such difference between male and female basketball athletes. The findings of this study indicate that the effects of long-term training in team ball sports transferred to improved visual attention and minimized sex differences in visual attention as assessed by tracking accuracy.

## Data availability statement

The original contributions presented in this study are included in the article/Supplementary material, further inquiries can be directed to the corresponding author.

## Ethics statement

The studies involving human participants were reviewed and approved by the Ethics Committee of the Shanghai University of Sport. The patients/participants provided their written informed consent to participate in this study.

## Author contributions

PJ and ZG contributed to the design of the study, data collection, and writing—original draft preparation. TF contributed to methodology and manuscript preparation. All authors read and agreed with the submitted version of the manuscript.

## Supplementary material

Supplementary Table 1 The raw data of tracking accuracy.
